# Work of the Red Cross

**Published:** 1923-10

**Authors:** 


### REPORT OF THE BRITISH RED CROSS.

The third Annual Report of the Joint Council of the Order of St. John of Jerusalem and the British Red Cross Society states that the decision to reorganise Departments at Headquarters with a view to greater economy in management, is reflected in the accounts. The total administrative expenditure for the past year on Departments other than stores, is over £3,000 less than for the year ended March 31, 1922. Considerable saving has been effected in rental charges and staff by the removal of the Stores Department from Whitfield Street to Headquarters. The Stores administration expenses for the past year are under £2,000, as compared with over £4,000 for the previous year. The work of the Ambulance service has grown considerably, and 326 ambulances are now in use. The Emergency Help Fund has assisted over 20,000 new cases, and the total cases dealt with at the Auxiliary Hospital for Officers was 635. As much as £13,000 was spent in providing for the treatment and general relief of tuberculous patients.

